# Association of host proteins with the broad host range filamentous phage NgoΦ6 of *Neisseria gonorrhoeae*

**DOI:** 10.1371/journal.pone.0240579

**Published:** 2020-10-15

**Authors:** A. Piekarowicz, A. Kłyż, M. Adamczyk-Popławska, D. C. Stein

**Affiliations:** 1 Department of Virology, Institute of Microbiology, Faculty of Biology, University of Warsaw, Warsaw, Poland; 2 Department of Cell Biology and Molecular Genetics, University of Maryland, College Park, MD, United States of America; Emory University School of Medicine, UNITED STATES

## Abstract

All *Neisseria gonorrhoeae* strains contain multiple copies of integrated filamentous phage genomes with undefined structures. In this study, we sought to characterize the capsid proteins of filamentous *N*. *gonorrhoeae* bacteriophage NgoΦ6 and phagemids propagated in different bacteria. The data demonstrate that purified phage contain phage-encoded structural proteins and bacterial host proteins; host proteins consistently copurified with the phage particles. The bacterial host proteins associated with the phage filament (as identified by mass spectrometry) tended to be one of the predominant outer membrane components of the host strain, plus minor additional host proteins. We were able to copurify a functional ß-lactamase, a phagemid-encoded protein, with phage filaments. We used protein modeling and immunological analysis to identify the major phage encoded structural proteins. The antigenic properties of these proteins depended on the bacterium where the phages were propagated. Polyclonal antibodies against *N*. *gonorrhoeae* phage NgoΦ6 recognized phage-encoded proteins if the phage was propagated in *N*. *gonorrhoeae* or *H*. *influenzae* cells but not if it was propagated in *Salmonella* or *E*. *coli*. We show that the phage filaments isolated from gonococci and *Haemophilus* are glycosylated, and this may explain the antigenic diversity seen. Taken en toto, the data demonstrate that while the neisserial filamentous phage are similar to other *Inovirus* with respect to overall genomic organization, their ability to closely associate with host proteins suggests that they have unique surface properties and are secreted by a here-to-fore unknown secretory pathway.

## Introduction

Bacteriophages of the genus *Inovirus* infect mostly Gram-negative bacteria, and their capsid is in the form of a long filamentous structure (750-nm to 3,000-nm) [1–5]. They are divided into two subgroups: (i) those able to integrate into the bacterial genome, like CTXΦ (*Vibrio cholerae* [6]), YpfΦ (*Yersinia pestis* [7]), ΦRSM (*Ralstonia solanaceum* [8]), MDAΦ (*Neisseria meningitidis* [9]) and NgoΦ6 (*N*. *gonorrhoeae* [10–12]) and (ii) those unable to integrate, represented among others by the bacteriophages of *Escherichia coli*, such as fd, f2, and M13 [1, 2]. The genomes of all of these phages are organized into three modules in which functionally related genes are grouped together [1, 13]; the replication genes (*gII*, *gV*, and *gX*); the structural genes (*gIII*, *gVI*, *gVII*, *gVIII*, and *gIX*); and the assembly and release genes (*gI* and *gIV*). Among the structural genes, *gIII* (or its equivalent) encodes the host recognition and adsorption protein. The key protein in the assembly and release module, pIV, produces an aqueous channel in the outer membrane through which phage particles exit from the host cell.

The filamentous virion structure is very simple, composed of a small number of proteins and ssDNA. In the Ff family of *Inoviruses*, thousands of helically arranged copies of pVIII (or an equivalent protein) form the filament tube. The type I signal sequence present in the C-terminal transmembrane helix of this protein allows for its secretion by SecYEG translocones, and YidC [4, 14] into to the inner membrane. The four minor proteins are all integral membrane proteins of which, only the pIII has a type I signal sequence [4, 15]. A few copies of “minor” phage proteins can play an essential role during infection and/or extrusion of the virion and are located at the end of the virion. The filamentous phage virion and assembly proteins belong to the cell secretome and which are secreted from bacteria without killing the host (for a recent review of the process, see [5]). The assembly is initiated by minor proteins pVII and pIX interacting with phage genome packaging signal followed by rapid elongation by addition of major coat (pVIII) subunits [3]. Most secreted proteins, among them the main structural proteins of filamentous phages, contain N-terminal signal sequences that are cleavable. Although the function and overall structure of the signal sequence and transmembrane alpha-helix are conserved in all domains of life, they lack primary sequence homology [4].

All *N*. *gonorrhoeae* genomes characterized to date encode several filamentous phages whose DNA and protein sequences have around ~95% [10, 11] identity at the nucleic acid level. Characterization of these DNA sequences allowed us to construct phagemids containing whole genomes of NgoΦ6 or NgoΦ7 using different plasmid backbones [11]. These phagemids were able to integrate into the host chromosome or be present as autonomous units and propagate in different gram-negative bacteria. The genetic organization of the NgoΦ6 and NgoΦ7 phage genomes possess the same blocks of genes responsible for particular phage functions as in Ff family of phages [11]. Among these blocks, it was predicted that the genes *orf3* to *orf6* are responsible for coding the structural proteins while *orf7* encodes a protein of 58 kDa that is a homolog of filamentous phage proteins responsible for adsorption to host cells. However, we have shown that an NgoΦ6 *orf7* deficient mutant is fully able to infect different bacterial genera [12] indicating that this phage can infect bacteria by different route than other *Inoviridae* and that Orf7 can play different role in biology of NgoΦ6. The ability to replicate, assemble and release from different types of gram-negative bacteria also suggests the use of a different mechanism than used by other filamentous phages.

In this paper, we demonstrate that the filament of NgoΦ6 phage and NgoΦ6 based phagemid particles contains not only bacteriophage encoded structural proteins but also copurifies with different host proteins (generally outer membrane proteins), depending on the host. The presence of such proteins in strong association with phage particles can explain their ability to infect, propagate and release from a broad group of gram-negative bacteria.

## Materials and methods

### Bacterial strains, plasmids, phages, and growth conditions

*Escherichia coli* Top10, F− *mcr*A Δ(mrr-hsdRMS-mcrBC) φ80 Δlac ΔM15 ΔlacX74 deoR recA1 araD139 Δ(araA-leu) galU galK λs-rpsL endA1 nup (pBSKS::Φ6fm:(EC)), *E*. *coli* BL21(DE3) [F− ompT gal dcm lon hsdSB (rB− mB−) λ(DE3)] (Novagen) were grown in Luria-Bertani broth (LB) at 37°C or 25°C. *Salmonella enterica* sv. Typhimurium χ3987(pBSKS::Φ6fm)(ST) and *S*. *enterica* sv. Typhimurium χ3987 (pMPMT6::Φ6fm)(ST) were grown in Luria-Bertani broth (LB) in the presence of diaminopimelic acid (DAP) (100 μg/ml final concentration). *Haemophilus influenzae* strain Rd (pBSKS::Φ6fm)(Hin) was grown at 37°C in brain heart infusion (BHI; Difco) supplemented with 2 μg of NAD/ml and 10 μg/ml of hemin [16]. Construction of *E*. *coli*, *S*. *enterica* sv. Typhimurium and *H*. *influenzae* Rd carrying pBSKSΦ6fm phagemid was described previously [12]. *Neisseria gonorrhoeae* strain FA1090 (obtained from Dr. W. Shafer at Emory University, Atlanta, GA), and were grown in phosphate-buffered gonococcal medium (Difco) supplemented with 20 mM glucose and growth supplements [17] either in broth with the addition of 0.042% NaHCO_3_ or on agar at 37°C in an incubator with 5% CO_2_. Plasmid pBluescript KS(+) was purchased from MBI Thermo Scientific. Phagemid pBSKS::Φ6fm and pMPMT6::Φ6fm construction and properties were described previously [12]. Depending on the last bacterial host where phagemids were propagated the name of this host will be added to the name of phagemid, for example pBSKS::Φ6fm (ST) will show the phagemid propagated in *Salmonella* strain.

### Phage and phagemid particles preparation

Phage were purified in one of two ways. In first method, an overnight culture of the appropriate strain carrying phage or phagemid genome was diluted 50-fold into 1000 ml of growth media with shaking (New Brunswick, 200 rpm) at 37°C until the OD_600_ was 0.2. Mitomycin C was added to final concentration of 35 ng/ml and growth continued overnight. In the second method, the treatment with mitomycin C was omitted. Bacteria were removed by centrifugation (20 min at 5 000 rpm). The supernatant was not filtered through any filters since even filtration through 0.8 μm filters resulted in a recovery only about 0.01% of phage or phagemid particles as measured by the concentration of ssDNA in the phage suspension. The phage suspension was mixed with 1/3 volume of a solution containing 20% polyethylene glycol (PEG-8000) and 2.5 M NaCl and kept at 4°C overnight to precipitate the phage particles. The precipitate was collected by centrifugation (20 min, 8 000 rpm, GS rotor, Sorval centrifuge), suspended in 4 ml of 20 mM KPO_4_ buffer (pH 7.2) and centrifuged for 10 min at 4 000 rpm (SS34 rotor, Sorval centrifuge) to remove any nonspecific precipitate. The supernatant was then centrifuged at 18 000 rpm (SS34 rotor, Sorval centrifuge) for 120 min. These last steps were repeated once more. The supernatant was removed and the precipitate suspended in 2 ml of different buffers depending on the method of further purification. Purification of phage on Sephacryl 4B column as previously described [18]

After purification, protein and ssDNA concentrations were determined using a NanoDrop apparatus. Alternatively, the purification of phage particles on CsCl gradient was carried out as described [19].

### Immunological methods and western blotting

To visualize phage structural proteins, purified phage or phagemid particles were suspended in 30 to 50 μl of gel loading buffer and analyzed by 4.0–15% gradient SDS-PAGE gels (BioRad), followed by staining with Coomassie Brilliant Blue. For Western blot analysis the proteins were transferred into positively-charged nylon membrane (Roche), blocked with 4% (w/v) nonfat milk in TBS at 25°C for 16 h and then incubated with various types of antibodies suspended in TBS at 16 ^o^C overnight. After three washes with 20 ml of TBS buffer, the membranes were incubated with secondary antibodies at room temperature for 1 h. Secondary antibody was removed and the membranes washed four times for 5 min with TBS + 0.1% Tween-20 and once with TBS. Membrane were then soaked in 20 ml of detection buffer (AP, 0,1 M Tris-HCl pH 9,5; 0,1 M NaCl; 5 mM MgCl_2_ pH 9,5) containing 20 μl of NBT BCIP for 30 min at room temperature in darkness. The reaction was stopped by intensive washing of membrane with distilled water and drying. The following primary antibodies were used during studies: (1) Rabbit anti-*N*. *gonorrhoeae* polyclonal antibody (GenWay Biotech. Inc., San Diego, CA; (2) Mouse anti-*N*. *gonorrhoeae* monoclonal antibody [386/418] (Abcam, Cambridge, MA); (3) Mouse anti-Beta- Lactamase antibody [8A5.A10] (Abcam); (4) Mouse anti flag-Tag (DYKDDDK) monoclonal antibodies (FG4R) Thermo Scientific; (5) rabbit serum obtained after vaccination with *S*. *enterica* sv. Typhimurium containing pBSKSΦ6fm (Named SAB, This study); (6) rabbit serum obtained after subcutaneous vaccination with phagemid pBSKS::Φ6fm(ST) (named SFAB); (7) rabbit serum obtained after subcutaneous vaccination with phage particles isolated from *N*. *gonorrhoeae* (named GCAB) [20] and anti-His Tag (Abcam) (dilution 1:500). The following secondary antibodies were used: secondary mouse monoclonal 2A9 anti-rabbit IgG heavy b chain (Alkaline Phosphatase) (Abcam) (1:2000 dilution; and secondary goat anti-mouse IgG H&L (Alkaline Phosphatase (Abcam) (dilution 1:4000).

### Transformation

Transformation-competent *E*. *coli* and *Salmonella* cells were prepared according to a procedure described previously [21] and stored at −80°C. To prepare cells for transformation, cells were thawed on ice, DNA added, and the mixture incubated on ice for 10 min. The bacteria were heat shocked at 37°C for 2 min, the total volume in the tube was increased to 1 ml by the addition of LB broth, and the transformation mixture incubated at 37°C for 30 min to 1 h to allow the bacteria to recover and begin expressing antibiotic resistance proteins. Transformed bacteria were plated onto LB agar plates containing appropriate antibiotics.

### Detection of glycosylation of proteins

Detection of glycosylated proteins was carried out using Pro-Q EMERALD 488 method according to recommendation of manufactures (ThermoFisher Scientific).

### Bioinformatic analysis and protein modeling

Modeling of proteins was carried out using an online service provided by Protein Structure Bioinformatics Group, Swiss Institute of Bioinformatics, Biozentrum, University of Basel based on the following work: [22–25]. Sequence searches against the non-redundant protein sequence (nr) database to identify evolutionarily related proteins were performed using PSI-BLAST (NCBI). Expectation (E) values of <10^−3^ were considered to be indication of homology.

### Protein sequencing

To identify proteins associated with phage particles, phage preparations were run on gradient 4–16% SDS-PAGE gels, stained with Coomasie Blue and particular bands were cut from the gel. Proteins were reduced with ß-mercaptoethanol and degraded by treatment with trypsin. The resulting peptides were separated by liquid chromatography (LC) and measurements of molecular mass of the peptides and their fragments determined by Mass Spectrometry. Comparison of resultant molecular masses of peptides and their fragments was determined using a data base of proteins (NCBI, UniProt, using program MASCOT) (http://www.matrixscience.com/). Mass Spectroscopy was performed in the laboratory of IBB PAN in Warsaw (Institute of Biophysics and Biochemistry Polish Academy of Sciences).

### Determination of β-lactamase using nitrocefin disk

Nitrocefin disks for microbiology were obtained from Sigma-Aldrich (49862 MSDS) and used according to manufactures instructions.

## Results

### Proteins present in phage and phagemid NgoΦ6 particles

**Fig 1:**
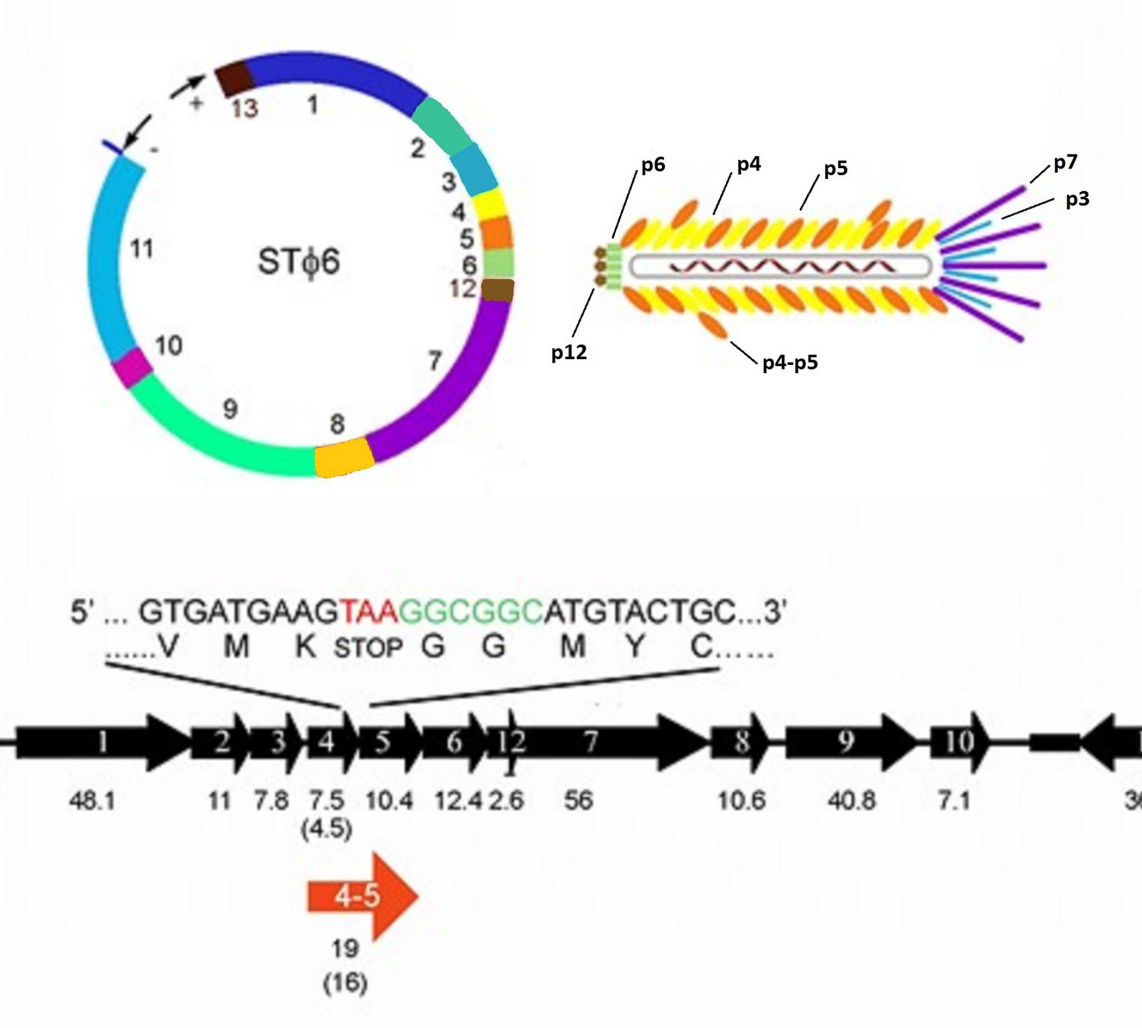
Proposed structural model of NgoPhi6. Panel A depicts the structural organization of the phagemid pBSKS::Φ6fm(ST) as described by Piekarowicz et al [12]. The numbers in the inner circle indicate the open reading frames. The colors correspond to their proposed location in the mature phage particle. Panel B indicates the predicted size of each protein. The inset show the DNA sequence and coding sequence between Orf4 and Orf5. The arrow in red indicated the potential size of a fusion protein between Orf4 and Orf5.

**Fig 2:**
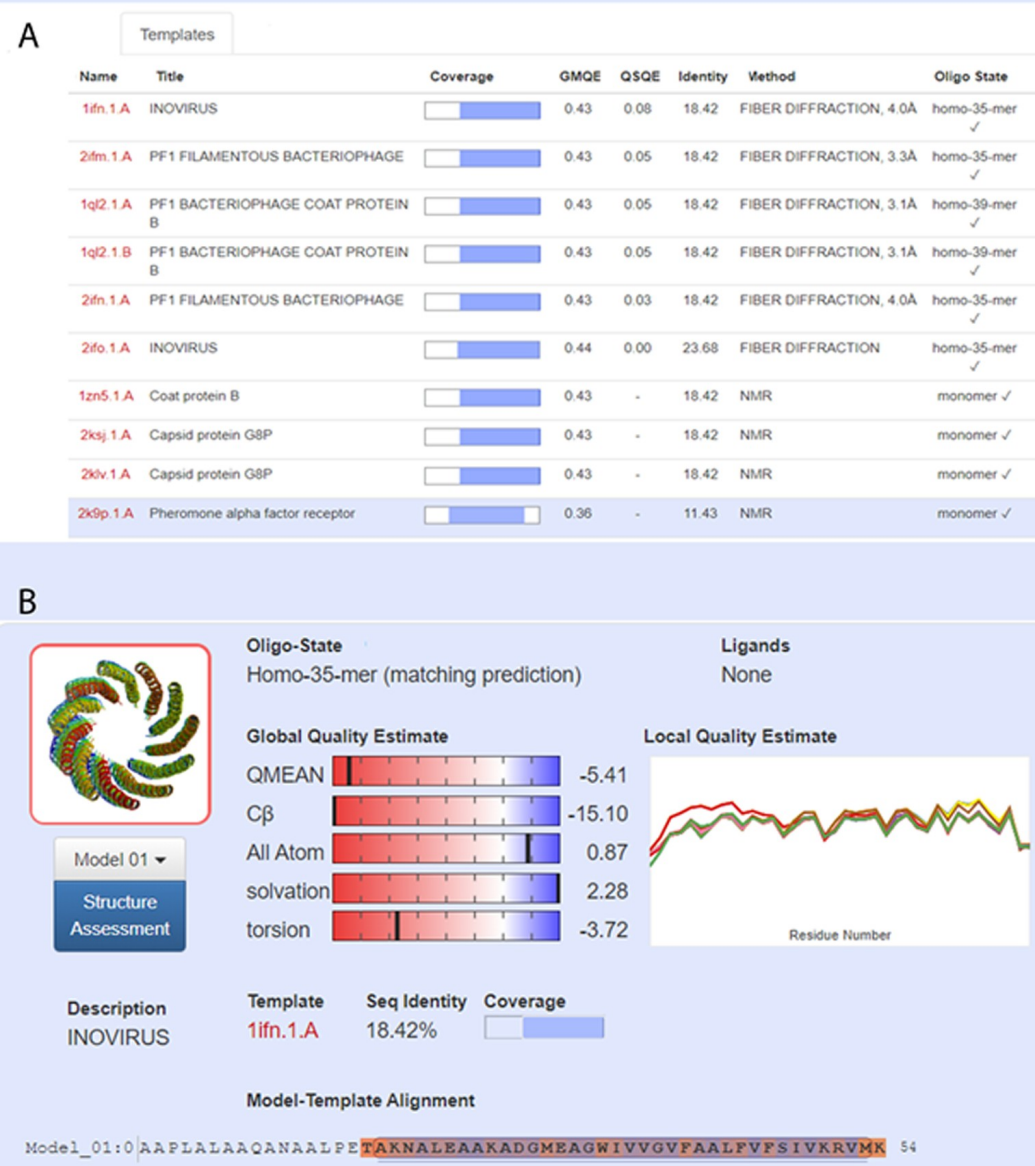
Protein modeling of Orf4. Modeling of proteins was carried out using an online service provided by Protein Structure Bioinformatics Group, Swiss Institute of Bioinformatics, Biozentrum, University of Basel as described in the materials and methods.

The major coat proteins of the *Inovirus* filamentous phages belong to four groups: Class I Ff group, Class I not Ff group, Class II and unclassified [4]. Among the 11 annotated open reading frames found in NgoΦ6, the genes encoding sequences Orf3-Orf7 (molecular size of 7.8 kDa, 7.5 kDa, 10.5 kDa, 2.6 kDa (Orf12), 12.5 kDa, and 58 kDa predicted from DNA sequence) are probably structural proteins (see Fig 1 for cartoon depiction of the organization). Because none of the presumptive structural proteins of NgoΦ6 showed amino acid sequence homology to any of the *Inovirus* major coat proteins with BLASTp (i.e P8 of M13), we searched the SWISS-MODEL template library SMTL version 2020-03-18, PDB release 2020-03-13 with BLAST [26] and HHBlits [27] for evolutionary related structures to these proteins. This modeling program provides a variety of quantitative readouts, which defines the relationship of the input protein to the comparator. QMEAN, which stands for Qualitative Model Energy ANalysis, is a composite scoring function describing the major geometrical aspects of protein structures. Five different structural descriptors are used [28]. In the top part of Fig 2, the data indicate that The majority of the amino acid sequence of Orf4 has significant similarity with other structural proteins of the *Inovirus* family, The low overall QMEAN score gives confidence that Orf 4 is structurally quite similar to the Inovirus structural proteins. This modeling of Orf4 showed that after excluding the 5’ leader sequence it had strong characteristics of main *Inovirus* filamentous phage coat proteins (Fig 2). This modeling data also suggested that this protein would self oligomerize. Modeling of Orf5 also showed very limited structural homology to these proteins at the C-end of Orf5 (See S1 and S2 Figs for complete data reports). This type of modeling suggests that Orf4 is the main structural phage encoded protein, and Orf5 is an accessory structural protein in NgoΦ6.

Because filamentous phage purified from *N*. *gonorrhoeae* could be a mixture of NgoΦ6, NgoΦ7, NgoΦ8 and NgoΦ9 proteins, we use phagemid derivatives of phage NgoΦ6 introduced into several species of bacteria that we previously described [12] to characterize proteins associated with phage filaments. We used phagemid purified from *S*. *enterica* sv. Thyphimurium carrying pBSKS::Φ6fm(ST) and pMPMT6::Φ6fm(ST). These strains were obtained by transformation of pBSKS:Φ6fm(EC) and pMPMT6::6fm(EC).

**Fig 3:**
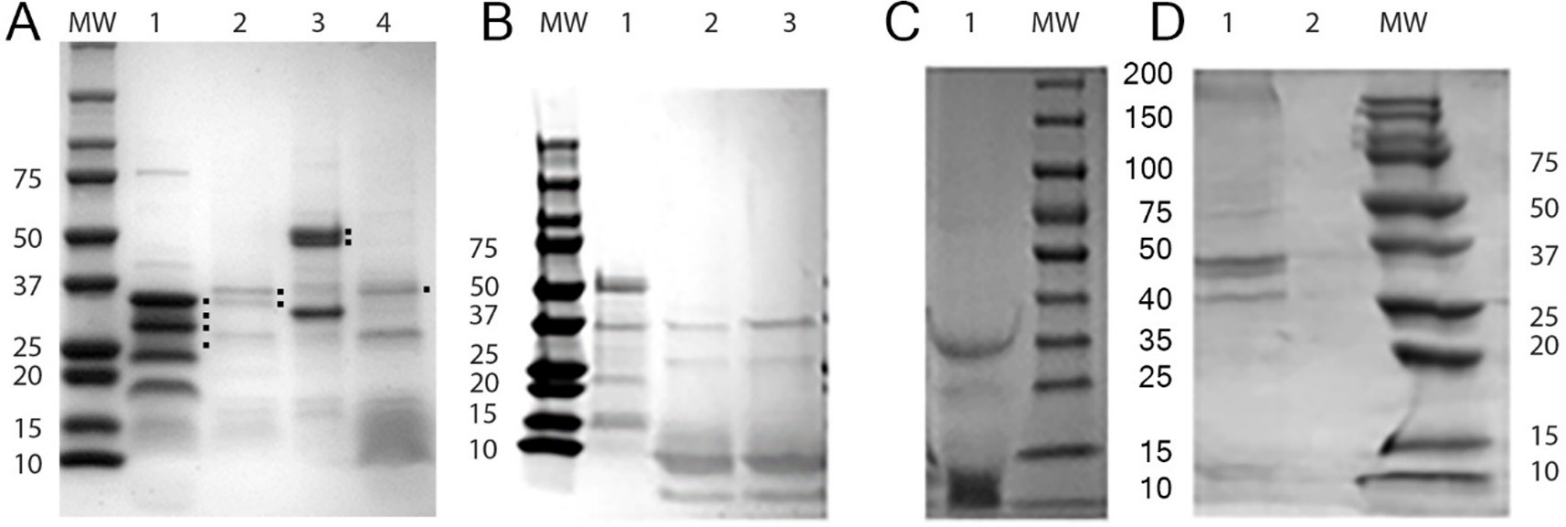
Proteins contained in phage/phagemids isolated from various bacterial strains. Filamentous phage NgoΦ6 of *N*. *gonorrhoeae* or its phagemid derivatives were purified by various methods: (Panel A) Purification by PEG/NaCl precipitation and two rounds of centrifugation at 38 000 g; (Panel B) centrifugation in CsCl gradients; (Panel C), purification on Sephacryl 4B column and (Panel D) *S*. *enterica* cells not carrying any phagemid genome where phagemid particles were isolated in parallel from *S*. *enterica* ser.Thyphimurium carrying pBSKS::Phi6fm (lane 1 and not carrying phagemid genome (lane 2). Proteins were separated on a 5% to 16% SDS-PAGE gel, and then stained with Coomassie Brilliant Blue. Panel A; lane 1: Phage NgoΦ6: lane 2, phagemid pMPMT6::Φ6fm isolated from *E*. *coli* strain DH5 alfa mcr); lane 3, phagemid pMPMT6::Φ6fm isolated from *S*. *enterica* sv. Typhimurium; and lane 4, phagemid pBSKS::Φ6fm isolated from *H*. *influenzae*. This figure is part of a previously published supplemental figure [30]. Panel B; lane 1, phagemid pBSKS::Φfm(ST) without induction with mitomycin C, lane 2, phagemidΦ pBSKS::Φ6fm(EC) without induction with mitomycin C, lane 3, phagemid pBSKS::Φ6fm(EC) after induction with mitomycin purified on Sephacryl 4B, Panel D, lane 1, phagemid pBSKS::Φ6fm(EC), lane 2, protein extract from *E*. *coli* cells not carrying any phagemid genome. Black dots on the right side of protein bands in Panel A identify protein used for their identification by Mass Spec; 35 kDa, 29 kDa, 26 kDa. 25 kDa from NgoΦ6; 38 kDa, 37 kDa and 29 kDa from pMPMT6::Φ6:fm(EC); 52 kDa and 51 kDa from pPMPMT6::Φ6fm (ST); 39 kDa from pBSKS::Φ6fm(HI).

Because the method of phage induction can impact the expression of host cell/phage proteins [29], we tested whether the induction by mitomycin C of phage/phagemid or the purification method employed influence the contents of proteins associated with phage particles. Phagemids were purified and their protein content analyzed on SDS-PAGE gel. The data (Fig 3, Panel A) show the presence of several low molecular weight bands, which should represent the major structural proteins of the phage (Because there are several forms of these proteins, the multiple bands do not resolve well in the 10–15 kDa range on this gel). However, the majority of visible bands did not correspond to bands with the predicted molecular weight of phage proteins, and the overall profiles differed significantly, depending on the host species from which the phage/phagemids were isolated. For example, the prominent bands associated with phage filaments isolated from phage NgoΦ6 are all in the 20–37 KDa range, while the predominate bands isolated from phagemid obtained from *Salmonella* are in the 37 to 50 kDa range. These variable profiles were consistently obtained, even after purification of phage by CsCl centrifugation, or after independent purification on different days (see S6 Fig for images of SDS profiles obtained with independent purification protocols/days. The data in Fig 3, panel B show that induction with mitomycin C did not alter the phage protein profiles obtained with phagemid isolated from *E*. *coli*. The method of purification did not impact the protein profiles. Compare Fig 3, panel C lane 1 (Sephacryl 4B purification) vs panel B lane 3 (CsCl purification).

We estimated the titer of phage by determining the concentration of sDNA in a particular phage preparation and normalizing this to the size of the phage genome. Using this measure, we usually obtained a phage titer of ~ 1x10^10^ particles/ml. To determine the number of protein molecules in NgoΦ6, we used densitometry to generate a profile of protein bands from an SDS-PAGE gel of purified NgoΦ6, adjusted the readings for molecular mass and titer of the preparation and determined that the virions contain about 780 molecules of 34.5 kDa protein, 450 of 30 kDa protein, 300 molecules of 26.0 kDa protein, 700 molecules of 18.0 kDa protein, 700 of 12.5 kDa protein and 650 of 7.5 kDa protein.). Similar results were obtained for phagemid based on phage NgoΦ6 (S1–S3 Tables).

### Phage host modifies the phage immunogenicity

**Fig 4:**
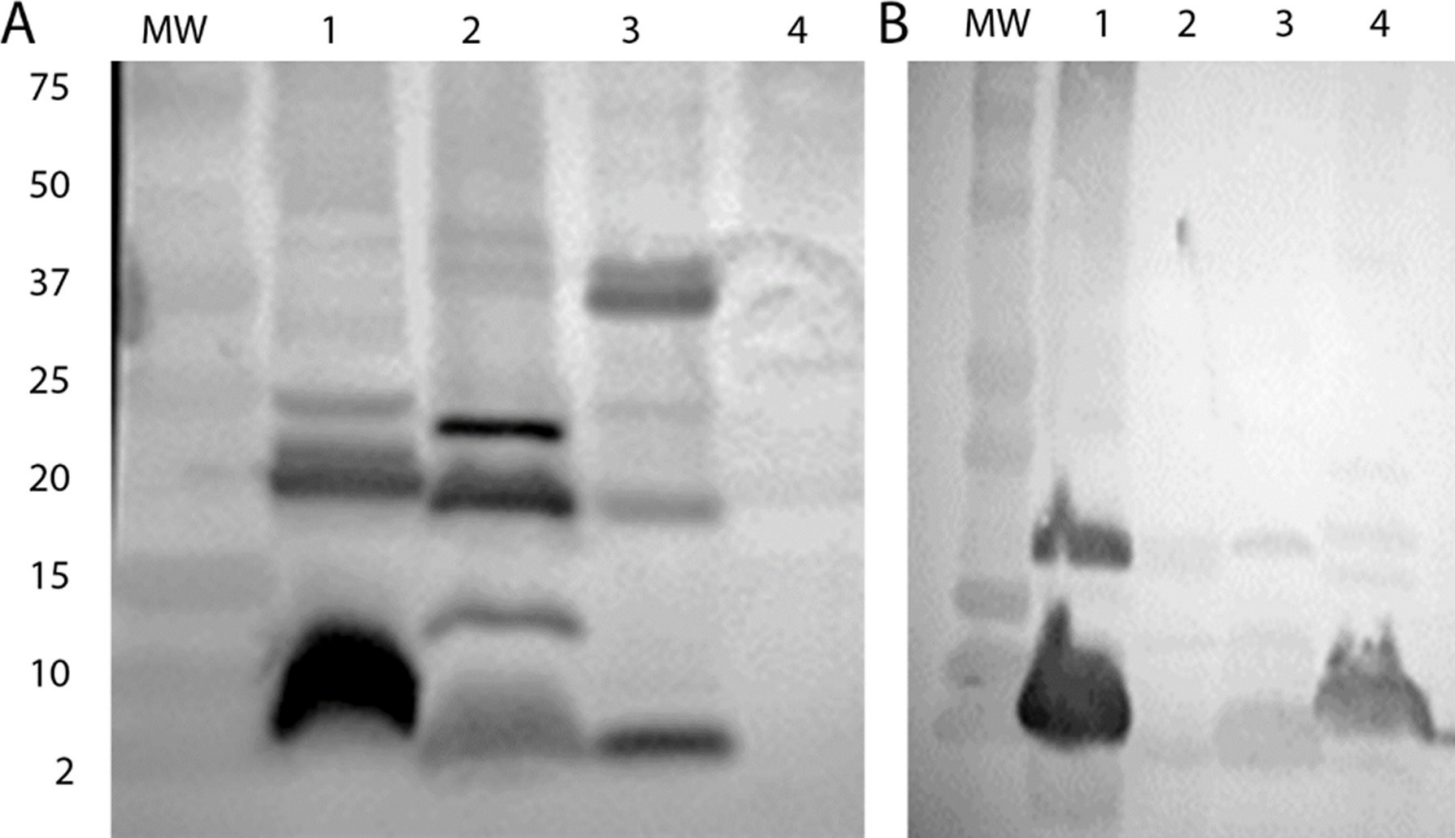
Impact of host strain on antibody reactivity. Phage NgoΦ6 and its phagemid derivatives were isolated and extracts separated on a 16% SDS-PAGE gel and subjected to Western blot analysis with antibodies that were elicited by immunization of rabbits with *S*. *enterica sv*. Typhimurium containing pBSKSΦ6fm (panel A) or with purified NgoΦ6 (Panel B The lanes in Panel A represent: 1) pBSKSΦ6::fm isolated from *H*. *influenzae*, 2) pBSKSΦ6::fm: isolated from *E*.*coli*; lane 3 pMPMT6Φ6::fm isolated from *Salmonella*, and lane 4) NgoΦ6. The lanes in Panel B represent: 1) NgoΦ6 2) pBSKSΦ6::fm isolated *from E*. *coli*; lane 3 pMPMT6Φ6::fm isolated from *S*. *enterica*; and lane 4) pBSKSΦ6::fm isolated from *H*. *influenzae*. Equal amounts (10 μg) of phage proteins were loaded on the gel.

**Fig 5:**
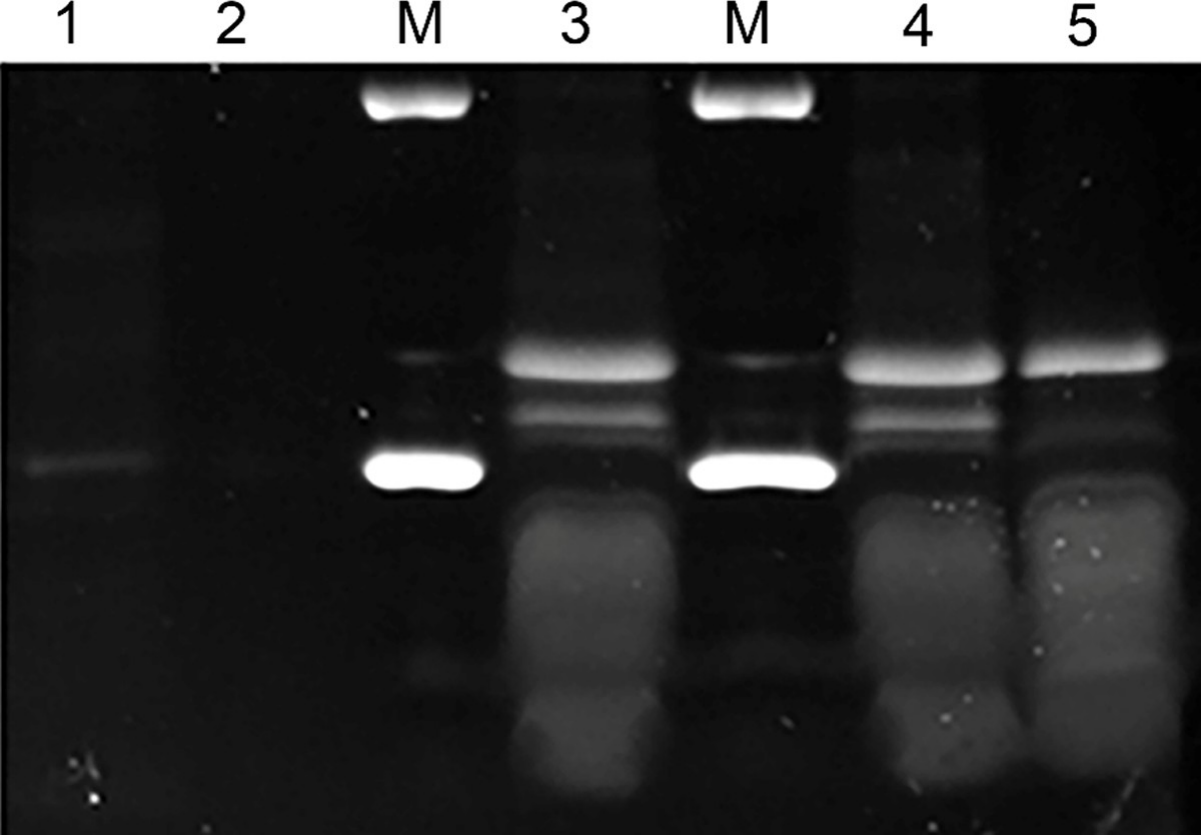
Identification of protein glycosylation. Proteins extracted from phage or phagemid particles were separated on a 5% to 15% SDS-PAGE gel and stained with Pro-Q Emerald 480 Glycoprotein Detection kit and then visualized with UV illumination. The lanes represent: MW, BioRad Dual Extra Molecular weight standards; Lane 1; phagemid pBSKS::Φ6fm isolated from *E*. Top10, lane 2; pBSKSΦ6::fm from *S*. *enterica*; lane 3 and 4; phage NgoΦ6; lane 5, pBSKS::Φ6fm isolated from *H*. *influenzae*. Equal amounts (10 μg) of phage proteins were loaded on the gel. The lanes M represent two proteins of molecular size 18 kDa and 42 kDa of CandyCane Glycoprotein Molecular Weight Standards stained by Emerald 488.

The antigenic properties of phage should be independent from the host used to propagate them. However, polyclonal antibodies elicited in rabbits immunized with filamentous phage propagated in *N*. *gonorrhoeae*, or *S*. *enterica sv*. Typhimurium show different reactivity with the same phage encoded proteins (see Fig 4). When antibody to the phagemid was raised against *S*. *enterica sv*. Typhimurium -propagated phagemids, reactivity was seen against several bands whose mass are consistent with phage structural proteins (Fig 5A, lane 1). However, this antisera was unable to bind to any proteins contained in NgoΦ6 (Fig 4A, lane 4). Conversely, anti-phage NgoΦ6 antibodies react with structural proteins of phage/phagemid propagated in *N*. *gonorrhoeae* (Fig 4B lane 1) and *H*. *influenzae* (Fig 4B, Lane 4) but not with phagemids propagated in *S*. *enterica sv*. Typhimurium and *E*. *coli* cells (Fig 4B, lanes 2 and 3).

One explanation for this observation is that phagemid associated host proteins alter how antigens are presented to the immune system. This could be by altering the three dimensional conformation slightly, masking certain epitopes or enhancing the presentation of immunodominant epitopes. A second explanation is that the amount of fusion protein made by Orf4 and Orf5 may differ, depending on the host cells translational efficiency. Another explanation is that NgoΦ6 phage/phagemid structural proteins are differentially modified in bacterial cells during phage propagation. One common type of posttranslational modification of proteins is glycosylation [31]. To determine if the phage/phagemids were differentially glycosylated, we used a protein glycosylation detection stain to determine if any phage-associated proteins were glycosylated. The results presented in Fig 5 demonstrate that that two proteins associated with NgoΦ6 are glycosylated in *N*. *gonorrhoeae* and *H*. *influenzae* cells but not when purified from *S*. *enterica sv*. Typhimurium or *E*. *coli*. The masses of these two proteins are consistent with the predicted masses of the fusion proteins between Orf4 and 5. The absence of lower molecular mass bands suggests that the predominant form of the structural protein is the fusion protein. The absence of glycosylation in the extracts from *E*. *coli* and *S*. *Typhimurium* indicate that the fusion protein is rarely made, or that these strains lack the ability to glycosylate these proteins.

### Host bacterial proteins in phage NgoΦ6 and its phagemid derivatives

**Fig 6:**
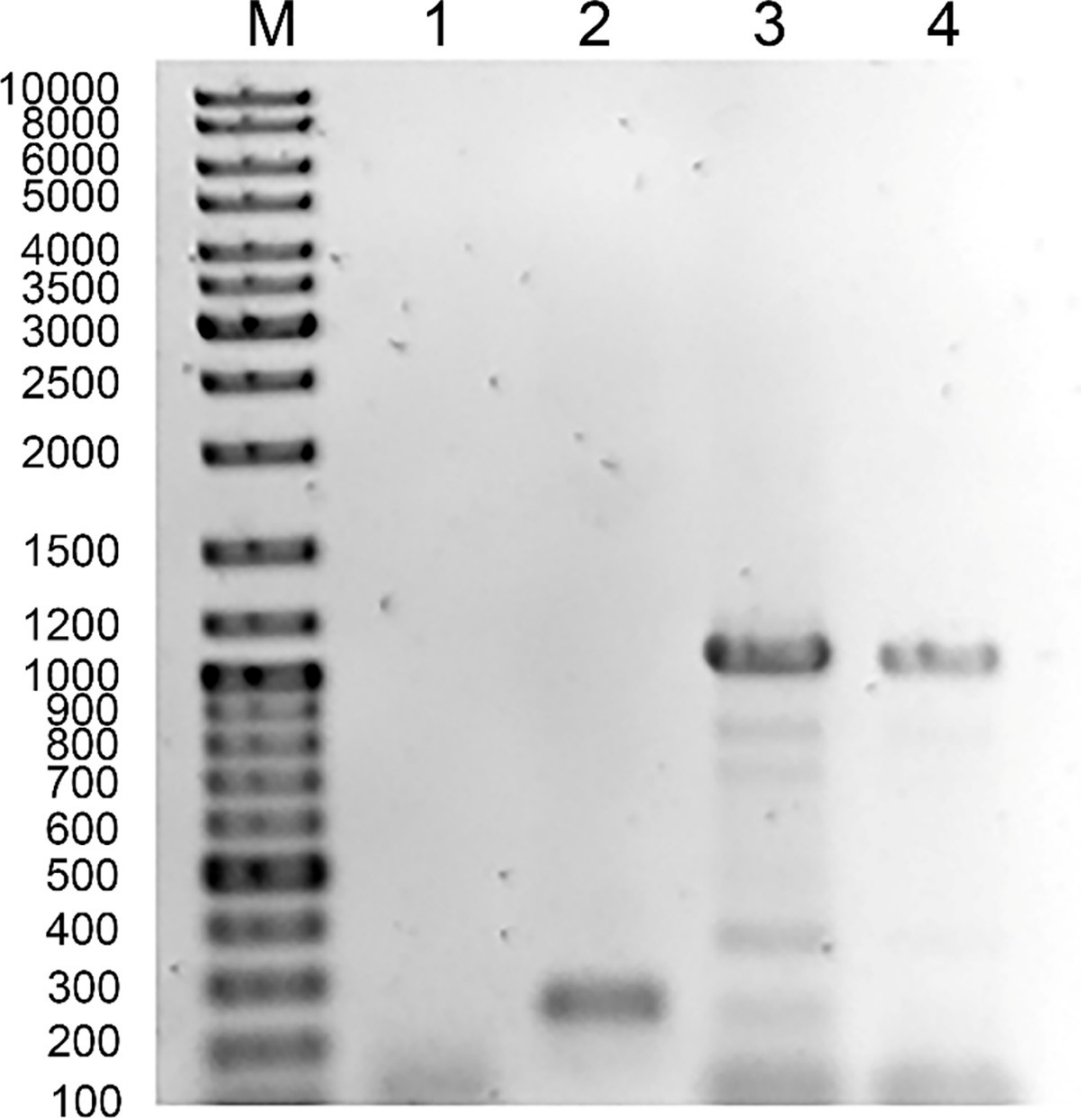
Phage do not contain host DNA. Lane 1 used DNA from NgoΦ6 phage particles purified after propagation in *N*. *gonorrhoeae* FA1090 cells. Lane 2; confirmation that NgoΦ6 DNA from NgoΦ6 phage particles contains phage genomic DNA; primers O4HisNF ATCATCACAAATTTATTAACACCTGCCG and O4HisNR GATGATGATGCATGATATTTTCCTTTACG were used to detect the presence of orf4 in phage particles purified after propagation in N. gonorrhoeae FA1090 cells. Lane 3 used DNA from phagemid particles pMPMT6::Φ6fm(EC) obtained from *E*. *coli* Top10 cells containing pET28::Por. Lane 4 used genomic *N*. *gonorrhoeae* FA1090 DNA as the source of DNA for PCR reaction. M;DNA ladder. Amplicons were obtained with primers PORFor CTAGCCTCTAGAATGAAAAAATCCCTGATTGCCCTG PORRev GATCCCGGGTTAGAATTTGTGGCGCAGAACGAC.

The SDS-PAGE analysis of the proteins of phage/phagemid particles propagated in *N*. *gonorrhoeae*, *S*. *enterica sv*. Typhimurium, *E*. *coli* and *H*. *influenzae* (Fig 3) showed different profiles. Even highly purified phage/phagemid particles propagated in *N*. *gonorrhoeae*, *S*. *enterica sv*. Typhimurium, *H*. *influenzae* and *E*. *coli* purified by on Sephacryl 4B column as previously described [18], on gradient of CsCl or PEG-NaCl precipitation and two cycles of centrifugation at 38 000 g and 4000 g showed the presence of non-phage encoded proteins with broad spectrum of molecular sizes. We hypothesized that these high molecular mass proteins represent host components. We excluded that the host DNA trapped in filamentous structure of phage encodes these proteins. The PCR analysis of the total DNA isolated from the phage particles grown in *N*. *gonorrhoeae* FA1090 did not show of the presence of DNA encoding Por protein (Fig 6) and previously we have shown that Ngoϕ6 or phagemids do not contain other type of host DNA [12].

To determine the identity of some of these high molecular mass proteins, phages were prepared, their proteins separated by SDS-PAGE, and the most prominent bands isolated from SDS-PAGE gel were analyzed by LC-MS-MS. The bands that were chosen for analysis are indicated in Fig 3. Database searches identified all of these (Table 1) as different surface proteins from the host cell, such as PorB and Opa from *N*. *gonorrhoeae*, flagellin from *S*. *enterica sv*. Typhimurium, and OmpA from *E*. *coli* (The complete data used to generate the data in Table 1 are included as S3–S5 Figs). These data suggest that as phage are extruded from the gonococcus, they incorporate outer membrane components. Because the mechanisms of phage replication in *S*. *enterica sv*. Typhimurium is unknown, the incorporation of flagellin into the phage particle suggests that it might be secreted by the flagellin biosynthesis machinery.

**Table 1:** Identification of cell proteins in phage/phagemid particles.

Bacteria	Phage/phagemid	Bands	Gi number	Protein	Mascot score	Number of peptide matches [aa]	Number of protein matches	Predicted mass
*N*.*gonorrhoeae* FA1090	NgoΦ6	1	Gi 170963340	Outer membrane protein (porin)	35882	695	18	35516
	NgoΦ6	2	Gi 59800533 Gi 59717428	Opacity protein B	1874	81	7	29626
	NgoΦ6	3	Gi 651851645	Outer membrane protein, (opacity protein)	3631	253	8	26529
	Ngo Φ6	4	Gi 651851637	Opacity Protein	10899	508	17	25997
*S*. *enterica* ser.ThyΦmirium	pMPMT6 Φ6	1	gi 315139288	phase-2 flagellin	2524	12	54	52915
	pMPMT6 Φ6	2	gi 346426943	Phase-1 flagellin	980	67	21	51581
*H*. *influenzae* RD KW20	pBS Φ6	1	gi 491963436	Outer membrane protein P2	447	9	34	39391
*E*. *coli* K12	pBS	1	gi 446788310	Outer membrane			24	13680
	pMPMT6	1		Outer membrane	1110	56	65	38234
	pMPMT6	2	gi16668 363					29031
	pMPMT6	3	446788310	Outer membrane protein (Por A)	551	12	25	37288

**Fig 7:**
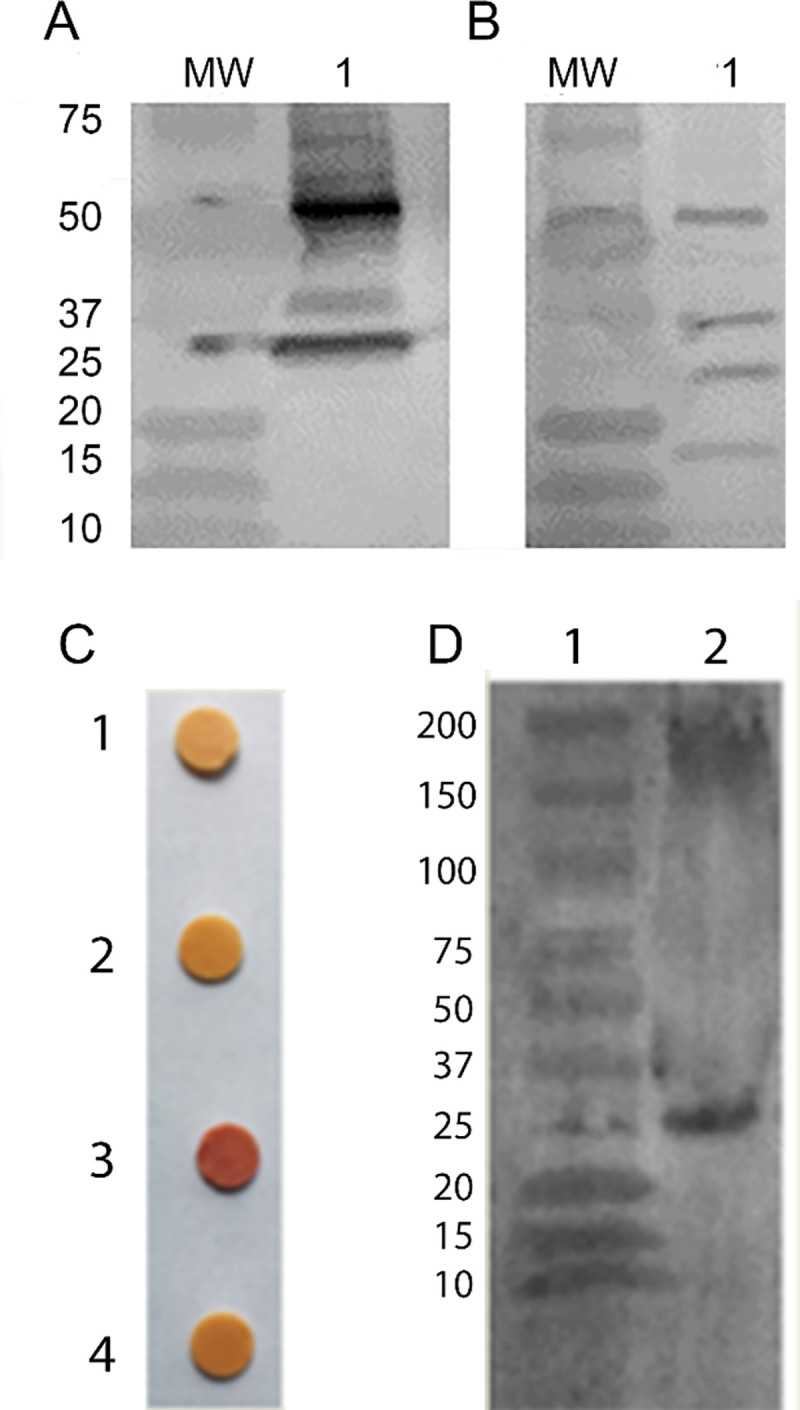
Detection of non-phage structural proteins in phage filaments. Phage/phagemid particles purified by PEG/NaCl precipitation followed by two cycle of centrifugation at 38 000 g and 4000 g were separated on 15% SDS-PAGE gels and subjected to Western blot analysis with commercial Abs. **Panel** A, lane 1, Reactivity of NgoΦ6 proteins with anti MPOM Abs, **Panel** B, lane 1, reactivity of pBSKSΦ6::fm: (ST) proteins with anti-flagella ABs. The presence of the TEM-2 β-lactamase in pBSKSΦ6::fm(ST) was demonstrated by preparing a phage suspension (20mg/ml protein) in 50 mM Tris buffer pH. 7.5 and spotting 10 μl on nitrocefin disc. Color was allowed to develop for 15 minutes and a change of color to pink or red was indicative of a positive reaction. In Panel C. the lanes represent: Disc 1, control containing buffer only; Disc 2, pBSKSΦ6:fm(EC); Disc 3, pMPMT6:Φ6fm(ST); and Disc 4, NgoΦ6 phage from *N*. *gonorrhoeae*. Panel D demonstrates the presence of ß-lactamase in phage particle. Phagemid particles were separated on SDS-PAGE gels and subjected to Western blot analysis Lane 1 is Molecular Weight Standards and Lane 2 is a western blot using monoclonal anti β-lactamase Abs.

The identity of some of these host proteins in NgoΦ6 was confirmed by reactivity with an antibody specific for Major principal outer membrane proteins (MPOM) (Fig 7A). The main host proteins present in pBSKSΦ6::fm (ST) identified as *S*. *enterica* flagella was confirmed by binding by polyclonal anti-*S*. *enterica* flagella antibodies (Fig 7B).

Because NgoΦ6fm can replicate in numerous species, we asked if it is possible to get particles carrying proteins not normally expressed in the host strain. The phagemid base for particle isolated from *S*. *enterica* encodes ß-lactamase while the phagemid isolated from *E*. coli encodes an aminoglycoside-modifying enzyme (acetyltransferase). The data in Fig 7, Panel C indicate that phagemid particles isolated from *S*. *enterica sv*. Typhimurium were able to cleave nitrocefin [32], while phagemids isolated from *E*. *coli* or phage isolated from *N*. *gonorrhoeae* could not. We further demonstrated that the phage particles, when analyzed by SDS-PAGE and Western blotting, contained a ß-lactamase (Fig 7, Panel D).

## Discussion

Structural models of fd bacteriophage as a representative model for class I filamentous phage have been generated by X-ray fiber diffraction and by solid-state NMR [13]. In these models, the fd particle (7 nm×880 nm) consists of a covalently closed, single-stranded DNA genome (6408 nucleotides) sheathed by 2750 copies of a 50 residues α-helical subunit (pVIII). These subunits are held together through hydrophobic interactions. The set ratio of subunits per nucleotide in Ff is 0.42 +/- 0.01 and 1 for Pf1 phage. The structural proteins of all filamentous phages studied to date are encoded by phage genomes and consists of the small number of proteins [33]. A few copies of “minor” phage proteins necessary for infection and/or extrusion of the virion are located at each end of each particle. While the overall genomic organization of the filamentous phage of *N*. *gonorrhoeae* would suggest a typical filamentous phages life cycle, phages NgoΦ6 and NgoΦ7 differ in several key aspects. They can replicate in broad group of Gram-negative bacteria [12] and they release mature phage particles and infect bacteria without the presence of Orf7 [30], an equivalent of pIII which in Ff filamentous phages play a role in assembly and releasing of mature virion from the membrane [13]. In this paper, we have demonstrated additional properties of these phage which may represent novel aspects in their biology.

While the genetic organization of the NgoΦ6/NgoΦ7 genome predict the presence of six presumable structural genes *orf3*, *orf4*, *orf5*, *orf12*, *orf6* and *orf7* [11], BLAST searches did not shown homology of these proteins to any other proteins encoded by filamentous phage. Our protein modeling data suggest that Orf4 has structural similarity to P8, the main structural protein of M13. Additionally, we have shown the presence of Orf4 and Orf5 in the structure of NgoΦ6 filament tube and the two fused forms of these proteins as the additional main structural protein [30]. We also show that host proteins can be incorporated into the phage filament.

If the basic filament structure of NgoΦ6 is formed by phage-encoded proteins and follows the same rules as for Ff filamentous phage, it would be difficult to imagine incorporation of the host proteins into this strictly formed rigid filamentous structure without distortion of the whole architecture. This suggests that the structure of the filament of NgoΦ6 and NgoΦ7 phage has to differ from classical structure of *Inoviruses* to allow for incorporation or strong association with host proteins.

The most striking property of the NgoΦ6 phage and its phagemid derivatives particles is the presence of the host proteins in the purified phage/phagemid preparations. Host proteins present in NgoΦ6 and its phagemid derivatives belong to different families of proteins whose common property is the presence of beta-barrel structures. The type of host protein depends on species of bacteria where phage/phagemid was propagated and is restricted only to its few members. The presence of the same host proteins in phage preparations is reproducible and did not depend on the phage purification method. In phagemid preparations, the specificity of host proteins does not depend on type of plasmid used for its construction. The incorporation of a functional ß-lactamase indicates that these proteins can retain their function, even when associated with the phage filament.

Our estimation of the number of structural proteins molecules per virion (3000–4000 nm long [34]) indicate that there are not enough molecules to form a structure similar to protein structure of Ff filaments. It is possible that NgoΦ6 encoded structural proteins form a scaffold that can be completed with host proteins. These proteins could be attached to the surface during their assembly and release from the cells.

What is the role of these cells proteins present in phage particles? Phage NgoΦ6, as well as NgoΦ7, can infect different types of Gram-negative bacteria [12] even without the presence of Orf7, the hypothetical phage receptor protein. Different types of bacterial proteins present in phage particles could recognize different bacterial cell proteins allowing tight contact between phage and the bacterial cell. For example, gonococcal Opa protein has been shown to bind to gonococcal lipooligosaccharide [35]. Its presence in NgoΦ6 phage particles could allow for enough association between the phage and the presumptive host to allow for injection of phage DNA across the membrane.

The difference between classical filamentous phages and NgoΦ6 lays also in their posttranslational modification. Staining of phage proteins with EMERALD 480 clearly showed that phage-encoded proteins present in phage particles are glycosylated when they were propagated in *N*. *gonorrhoeae*. Gonococcal glycoproteins share a number of intriguing features including their membrane tethering and translocation to or through the periplasm [36]. This would suggest that the antigenic properties of phage might differ, depending on the host strain from which they were propagated.

Without such modification phage encoded structural proteins should react with antibodies against phage particles independently of phage origin but instead the antibodies formed in rabbits vaccinated with *S*. *enterica* ser. Typhimurium or with pBSKSΦ6::ST do not recognize structural proteins of NgoΦ6 propagated in *N*. *gonorrhoeae* and vice versa. The same is true with bacterial proteins present in phage particles. *N*. *gonorrhoeae* proteins are recognized only by anti *N*. *gonorrhoeae* antibodies but not (or very weakly) by antibodies formed in rabbits vaccinated with *S*. *enterica* ser. Typhimurium what again can be connected to posttranslational modification.

Ff filamentous phages are released by a specific mechanism of assembly and secretion called extrusion. Filamentous phage assembly systems follow a variant of the two-step secretion process, where all virion proteins are first inserted into the inner membrane, followed by the export–coupled assembly of the phage. The assembly is initiated by minor’s proteins pVII and pIX that interact with a specific phage genome sequence called packaging signal followed elongation by addition of major coat pVIII protein. The assembly process is catalyzed by phage encoded inner membrane ATPase the filament is released from assembly site by two proteins pIII and pVI [3, 4, 37].

The release process of NgoΦ6 is not known but we previously showed that protein Orf9 located in the outer membrane play a role in this process [38]. Our results indicate that *N*. *gonorrhoeae* phage NgoΦ6 filament tube in contrast to other *Inoviruses* is differently constructed containing not only phage encoded structural protein but also host proteins originated mainly from bacterial outer membrane. An abundance of such protein in the host outer membrane would suggest incorporating them by chance during the assembly and release of NgoΦ6 phage. On the other hand, specificity and regularity in such process indicate that only some of them can be incorporated into phage particles.

The presence of host or even foreign proteins in the filament of NgoΦ6 and its phagemid derivatives has a lot of potential practical implications. Phagemids propagated in different bacterial strains incorporating host protein into the filament structure can be for their purification after disruption of phage structure. We have used this approach to purify Opa from gonococcal phage (AP, unpublished). They could also be used in different applications of phage display. Non host proteins or nonbacterial proteins present in the cell having beta-barrel structure can be intercalated into phage particles opening the way for new type of phage/cell protein display presentation.

## Supporting information

S1 Fig Swiss-model homology report for Orf 4. The Orf 4 coding sequence was used to generate this report.
(PDF)

S2 Fig Swiss-model homology report for Orf 5. The Orf 5 coding sequence was used to generate this report.
(PDF)

S3 Fig Proteomic analysis of major bands isolated from *N*. *gonorrohoeae* phage NgoΦ6. (JPG)

S4 Fig Proteomic analysis of major bands isolated from phagemid isolated from *E*. *coli*. (JPG)

S5 Fig Proteomic analysis of major banids isolated from phagemid isolated from *S*. *enteritica*. (JPG)

S6 Fig Reproducibility of proteins copurifying with phage. (DOCX)

S1 Table List of primers used for gene qRT-PCR. (DOCX)

S2 Table Number of copies of transcript per million copies of 16Sr RNA. (DOCX)

S3 Table Characterization of phage/phagemid preparations. (DOCX)
