# Supplementary material for: Association of host proteins with the broad host range filamentous phage NgoΦ6 of Neisseria gonorrhoeae
Source: PLoS One. 2020 Oct 15;15(10):e0240579. doi: 10.1371/journal.pone.0240579 (PMC7561177; doi:10.1371/journal.pone.0240579)
Supplement: S1 Fig — The Orf 4 coding sequence was used to generate this report. (PDF) [file pone.0240579.s001.pdf]

BIOZENTRUM University of Basel The Center for Molecular Life Sciences

SWISS-MODEL Homology Modelling Report

Model Building Report

This document lists the results for the homology modelling project "Untitled Project" submitted to SWISS-MODEL workspace on March 24, 2020, 8:08 p.m..The submitted primary amino acid sequence is given in Table T1.

If you use any results in your research, please cite the relevant publications:

- Waterhouse, A., Bertoni, M., Bienert, S., Studer, G., Tauriello, G., Gumienny, R., Heer, F.T., de Beer, T.A.P., Rempfer, C., Bordoli, L., Lepore, R., Schwede, T. SWISS-MODEL: homology modelling of protein structures and complexes. Nucleic Acids Res. 46(W1), W296-W303 (2018). doi>
- Guex, N., Peitsch, M.C., Schwede, T. Automated comparative protein structure modeling with SWISS-MODEL and Swiss-PdbViewer: A historical perspective. Electrophoresis 30, S162-S173 (2009). doi>
- Bienert, S., Waterhouse, A., de Beer, T.A.P., Tauriello, G., Studer, G., Bordoli, L., Schwede, T. The SWISS-MODEL Repository - new features and functionality. Nucleic Acids Res. 45, D313-D319 (2017). doi>
- Benkert, P., Biasini, M., Schwede, T. Toward the estimation of the absolute quality of individual protein structure models. Bioinformatics 27, 343-350 (2011). doi>
- Bertoni, M., Kiefer, F., Biasini, M., Bordoli, L., Schwede, T. Modeling protein quaternary structure of homo- and hetero-oligomers beyond binary interactions by homology. Scientific Reports 7 (2017). doi>

Results

The SWISS-MODEL template library (SMTL version 2020-03-18, PDB release 2020-03-13) was searched with BLAST (Camacho et al.) and HHBlits (Remmert et al.) for evolutionary related structures matching the target sequence in Table T1. For details on the template search, see Materials and Methods. Overall 23 templates were found (Table T2).

Models

No models have been built for this project.

Materials and Methods

Template Search

Template search with BLAST and HHBlits has been performed against the SWISS-MODEL template library (SMTL, last update: 2020-03-18, last included PDB release: 2020-03-13).

The target sequence was searched with BLAST against the primary amino acid sequence contained in the SMTL.

An initial HHblits profile has been built using the procedure outlined in (Remmert et al.), followed by 1 iteration of HHblits against NR20. The obtained profile has then be searched against all profiles of the SMTL. A total of 32 templates were found.

Model Building

Models are built based on the target-template alignment using ProMod3. Coordinates which are conserved between the target and the template are copied from the template to the model. Insertions and deletions are remodelled using a fragment library. Side chains are then rebuilt. Finally, the geometry of the resulting model is regularized by using a force field. In case loop modelling with ProMod3 fails, an alternative model is built with PROMOD-II (Guex et al.).

Model Quality Estimation

The global and per-residue model quality has been assessed using the QMEAN scoring function (Benkert et al.) . For improved performance, weights of the individual QMEAN terms have been trained specifically for SWISS-MODEL.

Ligand Modelling

Ligands present in the template structure are transferred by homology to the model when the following criteria are met: (a) The ligands are annotated as biologically relevant in the template library, (b) the ligand is in contact with the model, (c) the ligand is not clashing with the protein, (d) the residues in contact with the ligand are conserved between the target and the template. If any of these four criteria is not satisfied, a certain ligand will not be included in the model. The model summary includes information on why and which ligand has not been included.

Oligomeric State Conservation

The quaternary structure annotation of the template is used to model the target sequence in its oligomeric form. The method (Bertoni et al.) is based on a supervised machine learning algorithm, Support Vector Machines (SVM), which combines interface conservation, structural clustering, and other template features to provide a quaternary structure quality estimate (QSQE). The QSQE score is a number between 0 and 1, reflecting the expected accuracy of the interchain contacts for a model built based a given alignment and template. Higher numbers indicate higher reliability. This complements the GMQE score which estimates the accuracy of the tertiary structure of the resulting model.

References

- **BLAST**
  Camacho, C., Coulouris, G., Avagyan, V., Ma, N., Papadopoulos, J., Bealer, K., Madden, T.L. BLAST+: architecture and applications. BMC Bioinformatics 10, 421-430 (2009).
- **HHblits**
  Remmert, M., Biegert, A., Hauser, A., Söding, J. HHblits: lightning-fast iterative protein sequence searching by HMM-HMM alignment. Nat Methods 9, 173-175 (2012).

Table T1:

Primary amino acid sequence for which templates were searched and models were built.

```
MKFINTCRKYGAKLAVVTAAPLALAAQANAALPETAKNALEAAKADGMEAGWIVVGVFAALFVFSIVKRVMK
```

Table T2:

Template	Seq Identity	Oligo-state	QSQE	Found by	Method	Resolution	Seq Similarity	Coverage	Description
5tsa.1.A	16.98	monomer	-	HHblits	X-ray	2.40Å	0.27	0.74	Membrane protein
6pgi.1.A	16.98	monomer	-	HHblits	X-ray	3.50Å	0.27	0.74	BbZIP
6a7f.1.Q	27.78	homo-30-mer	0.11	HHblits	EM	NA	0.35	0.50	major coat protein p8
1ql2.1.A	18.42	homo-39-mer	-	HHblits	FIBER DIFFRACTION	3.10Å	0.32	0.53	PF1 BACTERIOPHAGE COAT PROTEIN B
1ql2.1.B	18.42	homo-39-mer	-	HHblits	FIBER DIFFRACTION	3.10Å	0.32	0.53	PF1 BACTERIOPHAGE COAT PROTEIN B
2ifm.1.A	18.42	homo-35-mer	0.01	HHblits	FIBER DIFFRACTION	3.30Å	0.32	0.53	PF1 FILAMENTOUS BACTERIOPHAGE
2klv.1.A	18.42	monomer	-	HHblits	NMR	NA	0.32	0.53	Capsid protein G8P
2ksj.1.A	18.42	monomer	-	HHblits	NMR	NA	0.32	0.53	Capsid protein G8P
1zn5.1.A	18.42	monomer	-	HHblits	NMR	NA	0.32	0.53	Coat protein B
1ifn.1.A	18.42	homo-35-mer	-	HHblits	FIBER DIFFRACTION	4.00Å	0.32	0.53	INOVIRUS
2ifn.1.A	18.42	homo-35-mer	-	HHblits	FIBER DIFFRACTION	4.00Å	0.32	0.53	PF1 FILAMENTOUS BACTERIOPHAGE
2mqw.1.A	19.05	monomer	-	HHblits	NMR	NA	0.29	0.58	26S proteasome regulatory subunit RPN9
2ifo.1.A	23.68	homo-35-mer	-	HHblits	FIBER DIFFRACTION	NA	0.32	0.53	INOVIRUS
1ifl.1.A	27.78	homo-55-mer	-	HHblits	FIBER DIFFRACTION	5.00Å	0.35	0.50	INOVIRUS
1ifp.1.A	20.59	homo-35-mer	-	HHblits	FIBER DIFFRACTION	3.10Å	0.30	0.47	MAJOR COAT PROTEIN ASSEMBLY
6kaf.1.N	32.35	monomer	-	HHblits	EM	NA	0.33	0.47	4.1 kDa photosystem II subunit
6kac.1.S	32.35	monomer	-	HHblits	EM	NA	0.33	0.47	4.1 kDa photosystem II subunit
6kad.1.r	32.35	monomer	-	HHblits	EM	NA	0.33	0.47	4.1 kDa photosystem II subunit

The table above shows the top 18 filtered templates. A further 5 templates were found which were considered to be less

suitable for modelling than the filtered list.
1pjf.1.A, 6tup.1.0, 1qzu.1.A, 3ifm.1.A, 1pfi.1.A
