# Supplementary material for: Association of host proteins with the broad host range filamentous phage NgoΦ6 of Neisseria gonorrhoeae
Source: PLoS One. 2020 Oct 15;15(10):e0240579. doi: 10.1371/journal.pone.0240579 (PMC7561177; doi:10.1371/journal.pone.0240579)
Supplement: S2 Fig — The Orf 5 coding sequence was used to generate this report. (PDF) [file pone.0240579.s002.pdf]

SWISS-MODEL Homology Modelling Report

Model Building Report

This document lists the results for the homology modelling project "Untitled Project" submitted to SWISS-MODEL workspace on March 24, 2020, 7:49 p.m..The submitted primary amino acid sequence is given in Table T1.

If you use any results in your research, please cite the relevant publications:

- Waterhouse, A., Bertoni, M., Bienert, S., Studer, G., Tauriello, G., Gumienny, R., Heer, F.T., de Beer, T.A.P., Rempfer, C., Bordoli, L., Lepore, R., Schwede, T. SWISS-MODEL: homology modelling of protein structures and complexes. Nucleic Acids Res. 46(W1), W296-W303 (2018). doi>
- Guex, N., Peitsch, M.C., Schwede, T. Automated comparative protein structure modeling with SWISS-MODEL and Swiss-PdbViewer: A historical perspective. Electrophoresis 30, S162-S173 (2009). doi>
- Bienert, S., Waterhouse, A., de Beer, T.A.P., Tauriello, G., Studer, G., Bordoli, L., Schwede, T. The SWISS-MODEL Repository - new features and functionality. Nucleic Acids Res. 45, D313-D319 (2017). doi>
- Benkert, P., Biasini, M., Schwede, T. Toward the estimation of the absolute quality of individual protein structure models. Bioinformatics 27, 343-350 (2011). doi>
- Bertoni, M., Kiefer, F., Biasini, M., Bordoli, L., Schwede, T. Modeling protein quaternary structure of homo- and hetero-oligomers beyond binary interactions by homology. Scientific Reports 7 (2017). doi>

Results

The SWISS-MODEL template library (SMTL version 2020-03-18, PDB release 2020-03-13) was searched with BLAST (Camacho et al.) and HHBlits (Remmert et al.) for evolutionary related structures matching the target sequence in Table T1. For details on the template search, see Materials and Methods. Overall 13 templates were found (Table T2).

Models

The following models were built (see Materials and Methods "Model Building"):

Model #02	File	Built with	Oligo-State	Ligands	GMQE	QMEAN
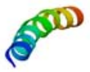	PDB	ProMod3 3.0.0	monomer (matching prediction)	None	0.11	-1.94

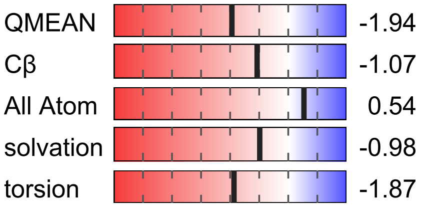

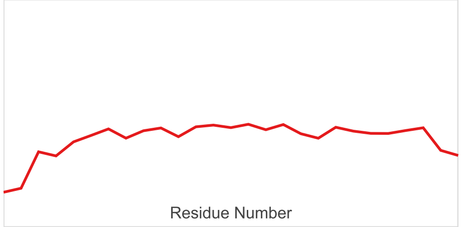

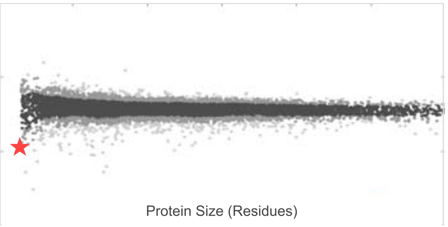

Template	Seq Identity	Oligo-state	QSQE	Found by	Method	Resolution	Seq Similarity	Range	Coverage	Description
2ifo.1.A	11.11	homo-35-mer	0.00	HHblits	FIBER DIFFRACTION	-	0.28	65 - 91	0.29	INOVIRUS

The template contained no ligands.

```
Target    MYCQVGNKCLEKHRAENLYFSLVVPRIKENGQIIRPEYNGSMWKMSDGQPLRLSLAECSPKDNLQSGLETGRIVFGVLAS
2ifo.1.A  ----------------------------------------------------------------GPIAAIGGAVLTVMVG

Target    VYFVSLLKKVLK
2ifo.1.A  IKVYKWVRRAM-
```

Model #01	File	Built with	Oligo-State	Ligands	GMQE	QMEAN
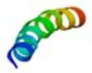	PDB	ProMod3 3.0.0	monomer (matching prediction)	None	0.11	-1.94

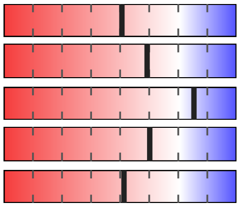

QMEAN		-1.94
Cβ		-1.07
All Atom		0.54
solvation		-0.98
torsion		-1.87

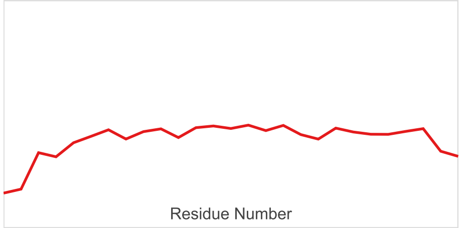

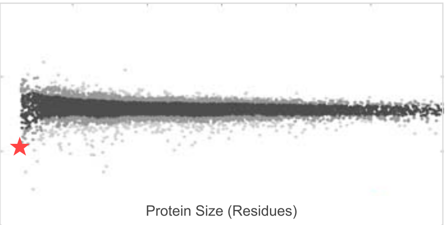

Template	Seq Identity	Oligo-state	QSQE	Found by	Method	Resolution	Seq Similarity	Range	Coverage	Description
2ifo.1.A	11.11	homo-35-mer	0.00	HHblits	FIBER DIFFRACTION	-	0.28	65 - 91	0.29	INOVIRUS

The template contained no ligands.

```
Target    MYCQVGNKCLEKHRAENLYFSLVVPRIKENGQIIRPEYNGSMWKMSDGQPLRLSLAECSPKDNLQSGLETGRIVFGVLAS
2ifo.1.A  ----------------------------------------------------------------GPIAAIGGAVLTVMVG

Target    VYFVSLLKKVLK
2ifo.1.A  IKVYKWVRRAM-
```

Materials and Methods

Template Search

Template search with BLAST and HHBlits has been performed against the SWISS-MODEL template library (SMTL, last update: 2020-03-18, last included PDB release: 2020-03-13).

The target sequence was searched with BLAST against the primary amino acid sequence contained in the SMTL.

An initial HHblits profile has been built using the procedure outlined in (Remmert et al.), followed by 1 iteration of HHblits against NR20. The obtained profile has then be searched against all profiles of the SMTL. A total of 13 templates were found.

Model Building

Models are built based on the target-template alignment using ProMod3. Coordinates which are conserved between the target and the template are copied from the template to the model. Insertions and deletions are remodelled using a fragment library. Side chains are then rebuilt. Finally, the geometry of the resulting model is regularized by using a force field. In case loop modelling with ProMod3 fails, an alternative model is built with PROMOD-II (Guex et al.).

Model Quality Estimation

The global and per-residue model quality has been assessed using the QMEAN scoring function (Benkert et al.) . For improved performance, weights of the individual QMEAN terms have been trained specifically for SWISS-MODEL.

Ligand Modelling

Ligands present in the template structure are transferred by homology to the model when the following criteria are met: (a) The ligands are annotated as biologically relevant in the template library, (b) the ligand is in contact with the model, (c) the

ligand is not clashing with the protein, (d) the residues in contact with the ligand are conserved between the target and the template. If any of these four criteria is not satisfied, a certain ligand will not be included in the model. The model summary includes information on why and which ligand has not been included.

Oligomeric State Conservation

The quaternary structure annotation of the template is used to model the target sequence in its oligomeric form. The method (Bertoni et al.) is based on a supervised machine learning algorithm, Support Vector Machines (SVM), which combines interface conservation, structural clustering, and other template features to provide a quaternary structure quality estimate (QSQE). The QSQE score is a number between 0 and 1, reflecting the expected accuracy of the interchain contacts for a model built based a given alignment and template. Higher numbers indicate higher reliability. This complements the GMQE score which estimates the accuracy of the tertiary structure of the resulting model.

References

- **BLAST**
  Camacho, C., Coulouris, G., Avagyan, V., Ma, N., Papadopoulos, J., Bealer, K., Madden, T.L. BLAST+: architecture and applications. BMC Bioinformatics 10, 421-430 (2009). doi>
- **HHblits**
  Remmert, M., Biegert, A., Hauser, A., Söding, J. HHblits: lightning-fast iterative protein sequence searching by HMM-HMM alignment. Nat Methods 9, 173-175 (2012). doi>

Table T1:

Primary amino acid sequence for which templates were searched and models were built.

```
MYCQVGNKCLEKHRAENLYFSLVVPRIKENGQIIRPEYNGSMWKMSDGQPLRLSLAECSPKDNLQSGLETGRIVFGVLASVYFVSLLKKVLK
```

Table T2:

Template	Seq Identity	Oligo-state	QSQE	Found by	Method	Resolution	Seq Similarity	Coverage	Description
1mhn.1.A	16.22	monomer	-	HHblits	X-ray	1.80Å	0.29	0.40	Survival motor neuron protein
4a4e.1.A	16.22	monomer	-	HHblits	NMR	NA	0.29	0.40	SURVIVAL MOTOR NEURON PROTEIN
4a4g.1.A	16.22	monomer	-	HHblits	NMR	NA	0.29	0.40	SURVIVAL MOTOR NEURON PROTEIN
2ifo.1.A	11.11	homo-35-mer	-	HHblits	FIBER DIFFRACTION	NA	0.28	0.29	INOVIRUS

The table above shows the top 4 filtered templates. A further 9 templates were found which were considered to be less suitable for modelling than the filtered list.
6qbj.1.A, 6olf.75.A, 6e0x.2.B, 6e0x.1.A, 6ole.76.A, 6olg.79.A, 6e0y.1.A, 1ifp.1.A, 6e17.1.A
