# Supplementary figures and images for: Association of host proteins with the broad host range filamentous phage NgoΦ6 of Neisseria gonorrhoeae
Source: PLoS One. 2020 Oct 15;15(10):e0240579. doi: 10.1371/journal.pone.0240579 (PMC7561177; doi:10.1371/journal.pone.0240579)

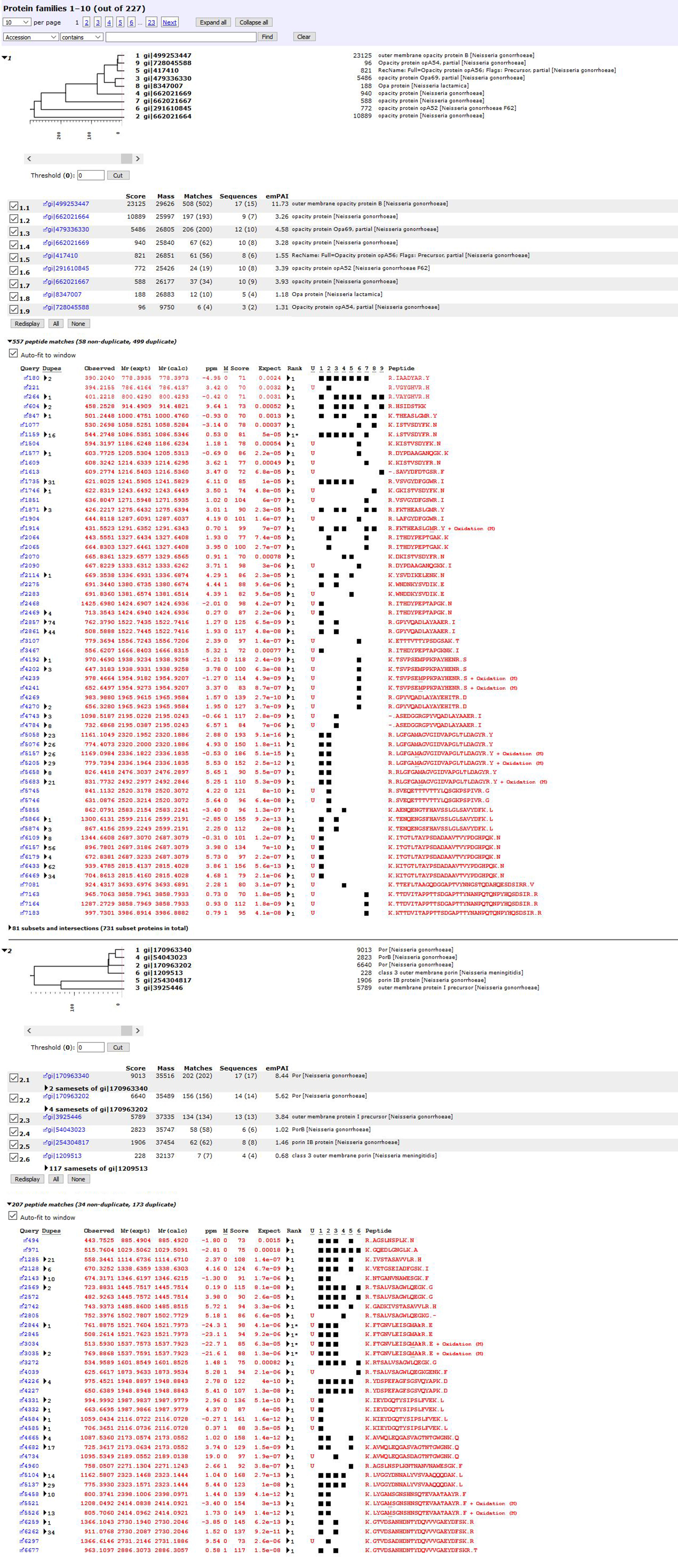

Supplement: S3 Fig — (JPG) [file pone.0240579.s003.jpg]

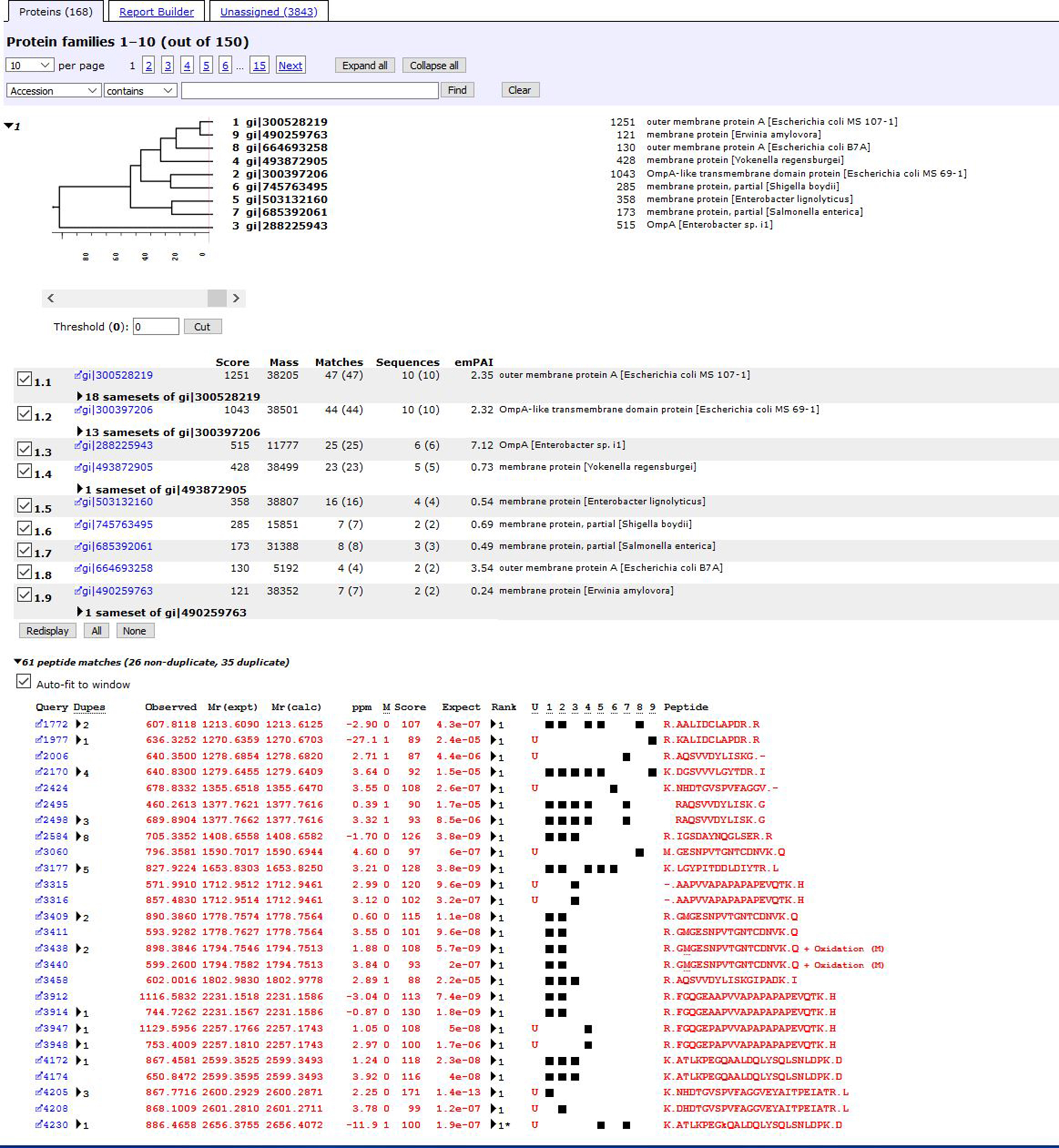

Supplement: S4 Fig — (JPG) [file pone.0240579.s004.jpg]

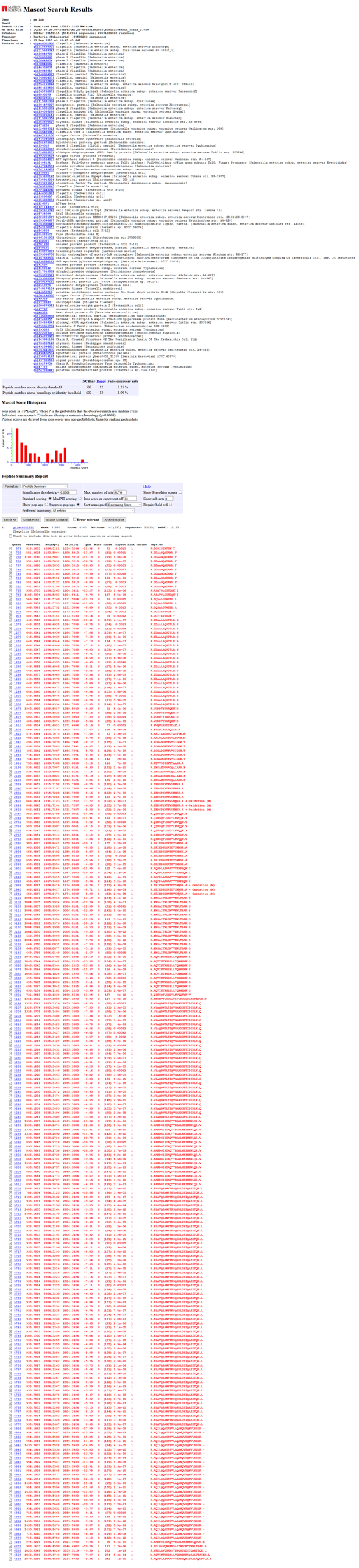

Supplement: S5 Fig — (JPG) [file pone.0240579.s005.jpg]
