# Supplementary material for: Association of host proteins with the broad host range filamentous phage NgoΦ6 of Neisseria gonorrhoeae
Source: PLoS One. 2020 Oct 15;15(10):e0240579. doi: 10.1371/journal.pone.0240579 (PMC7561177; doi:10.1371/journal.pone.0240579)

S6 Fig. Demonstration of reproducibility of protein association with phage filaments

Panel A. Phage/phagemid were purified by PEG/NaCl precipitation and two rounds of centrifugation at 38 000 g. The panel represents two independent purifications from the same bacterial culture (Panels A and B). The lanes represent proteins isolage from: 1) NgoΦ6 isolated from *N. gonorrhoeae* strain FA1090*;*  2) Phagemid pBSΦ6 isolated from *E. Coli*; 3) Phagemid pBSΦ6 isolated from  *Salmonella* and 4) Phagemid pBSΦ6 isolated from *H. Influenzae*. Panel B was used for paper


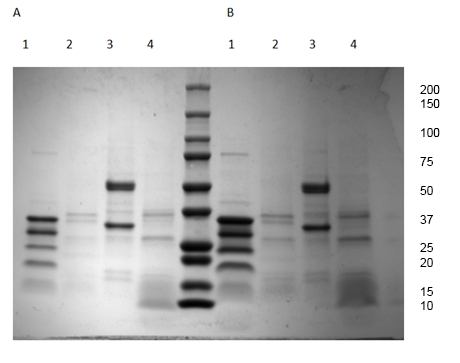


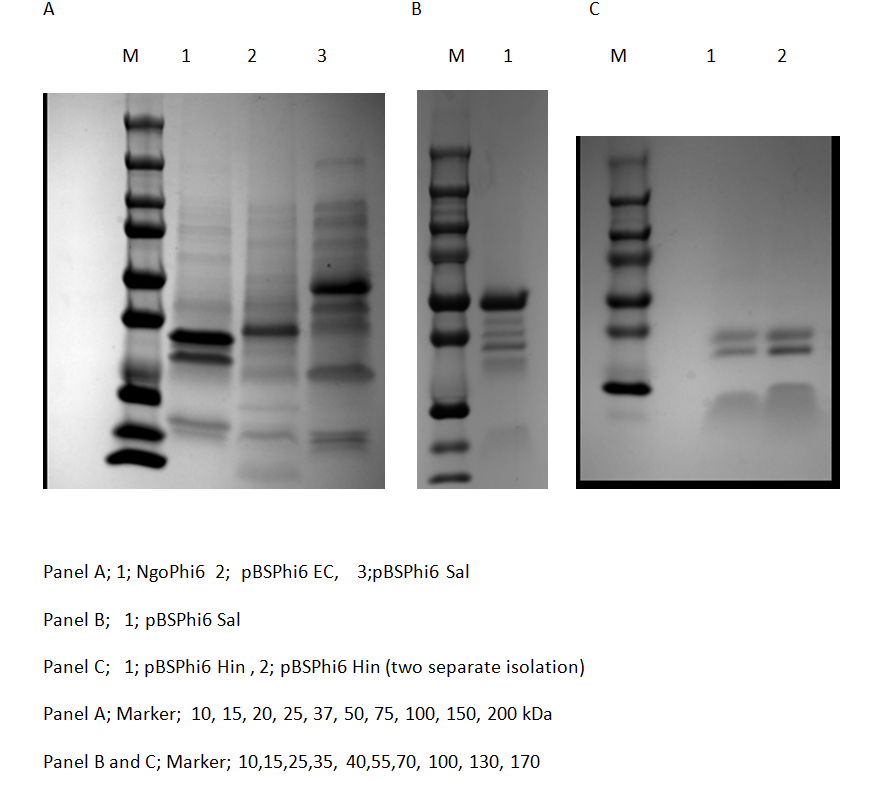


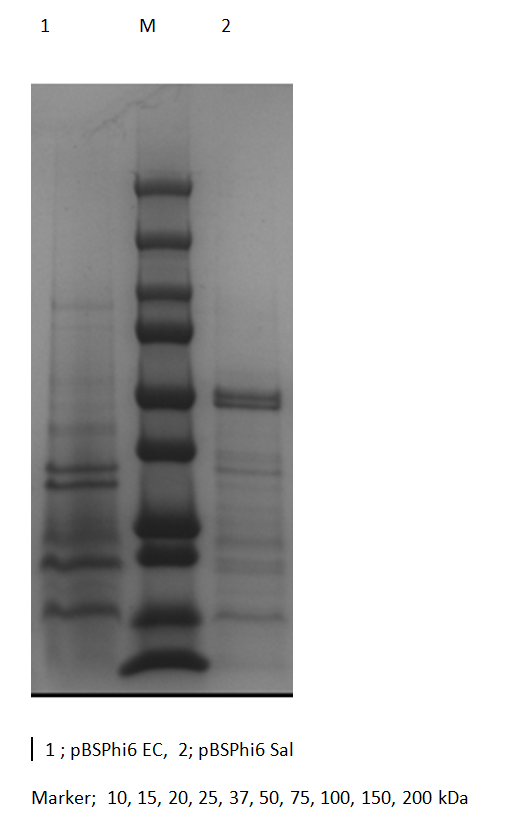


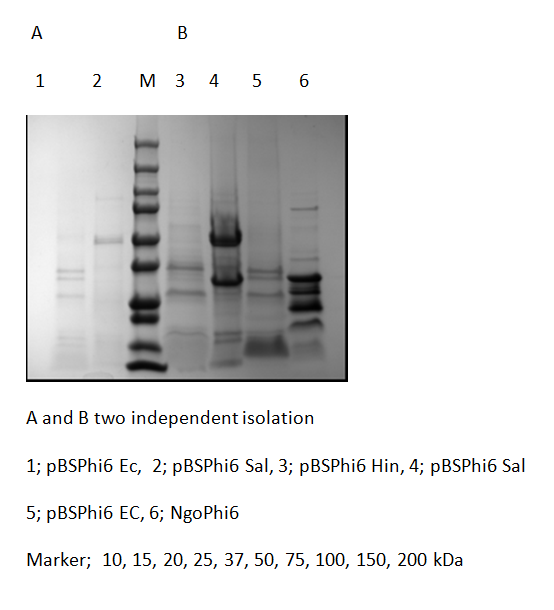


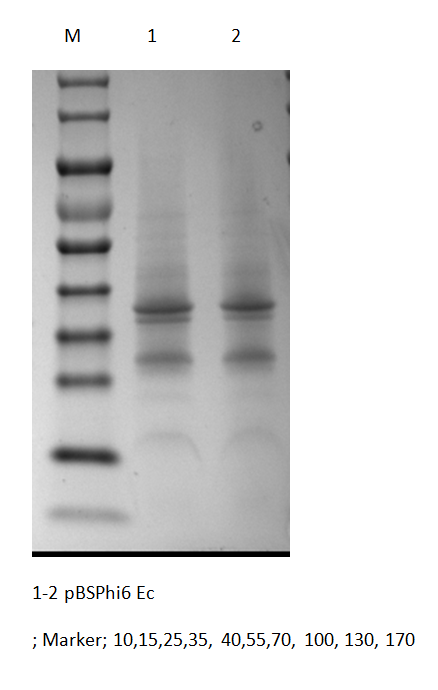

Supplement: S6 Fig — (DOCX) [file pone.0240579.s006.docx]
