# Supplementary material for: Association of host proteins with the broad host range filamentous phage NgoΦ6 of Neisseria gonorrhoeae
Source: PLoS One. 2020 Oct 15;15(10):e0240579. doi: 10.1371/journal.pone.0240579 (PMC7561177; doi:10.1371/journal.pone.0240579)
Supplement: S1 Table — (DOCX) [file pone.0240579.s007.docx]

**S1 Table. List of primers used for gene qRT-PCR.**

| Name^a^ | Forward primer | Reverse primer | Product size bp^d^ |
| --- | --- | --- | --- |
| 16SRNA^b^ | GCGTGGGTAGCAAACAGGAT | CGCGTTAGCTACGCTACCAAG | 101 |
| NGO1138 | ATTGGAAACCGCCTAAAGAA | TACAAACCCTACAACCGCAA | 116 |
| NGO1143 | GAAATTTATTAACACCTGCCGTAA | TTCCATACCGTCCGCTTT | 145 |
| NGO1142 | ATATAACGGCAGCATGTGGA | ACTATCCGGCCTGTTTCAAG | 111 |
| NGO1141 | GCAATCGATGGCGGTTA | CCAAATGTCATGTTTGATTCTTC | 121 |
| Int^c^ | TTCCATCGTTAAGAGGGTG | CCGGTGCTTCTCAAGACAT | 76 |
| ^a^Primer names are the same as target gene accession number unless otherwise noted. | | | |
| ^b^16SRNA primer specific for 16S rRNA genes. | | | |
| ^c^Int primer positioned at 3` end of NGO1143 and 5` of NGO1142 genes. | | | |
| ^d^Expected PCR amplicon size. | | | |
