# Supplementary material for: Association of host proteins with the broad host range filamentous phage NgoΦ6 of Neisseria gonorrhoeae
Source: PLoS One. 2020 Oct 15;15(10):e0240579. doi: 10.1371/journal.pone.0240579 (PMC7561177; doi:10.1371/journal.pone.0240579)
Supplement: S2 Table — (DOCX) [file pone.0240579.s008.docx]

**S2 Table. Number of copies of transcript per million copies of 16Sr RNA**

**Transcript 16SRNA orf1138a orf1143b orf1142c orf1142/orf1143d,g orf1141e**

**___________________________________________________________________________**

**Mean 1000000 25.47 4.13 2.65 11.74 37.04**

**SDh 0 7.56 1.86 0.44 0.98 ----___________________________________________________________________________**

**2a,orf b,c,d,e,f genes encoding hypothetical structural genes of phage NgoΦ6**

**g determination of the mRNA copies for fusion orf4 and orf5 genes allowing for formation of fusion ORF4 O RF5 protein**

**SDh; standard deviation**
