# Supplementary material for: Association of host proteins with the broad host range filamentous phage NgoΦ6 of Neisseria gonorrhoeae
Source: PLoS One. 2020 Oct 15;15(10):e0240579. doi: 10.1371/journal.pone.0240579 (PMC7561177; doi:10.1371/journal.pone.0240579)
Supplement: S3 Table — (DOCX) [file pone.0240579.s009.docx]

S3 Table. Characterization of phage/phagemid preparations.

___________________________________________________________________________

Phage/phagemid Concentration Concentration Number of ssDNA

of ssDNA of proteins molecules

(μg x 1 L-1) (mg x ml-1) 10^13^ x L-1

___________________________________________________________________________

Mean SD^a^ Mean SD^a^ Mean SD^a^

___________________________________________________________________________

NgoΦ6 358.33 22.54 11.02 0.69 8.22 0.51

pBSKS::Ngo

Φ6fm(EC) 238.33 107.74 6.33 3.23 5.1 3.02

pMPMT6::Ngo

Φ6fm(EC) 221.66 50.08 5.1 1.14 4.53 1.76

pBSKS::Ngo

Φ6fm(HI) 224.66 46.23 5.58 0.76 4.4 0.17

pBSKSNgo

Φ6fm(ST) 340.0 95.39 9.84 3.33 7.84 2.24

^a^ SD; standard devaition

The phage/phagemids were isolated from 1000 ml of appropriate culture without induction by mitomycin C and isolated by precipitation with PAGE-NaCl and double centrifugation at high and low speed as described in Materials and Methods. Finally the phage particles were suspended in 2 ml of SM buffer.

Determination of the number of particular proteins in phage preparation was done for *N. gonorrhoeae* filamentous phages. Since *N. gonorrhoeae* ds DNA phage particles Ngoφ1 and Ngoφ2 (1) are only present in culture supernatants after induction with mitomycin C, we assume that the purified filamentous phage preparations contains only those phages. Moreover, filamentous phagemid particles produced in *E. coli* or *Salmonella* strains are produced in strains free of any other phages and therefore cannot be contaminated by other phages. We do not have the method allowing for determination of filamentous phage titers by “plaques” method thus the number of phage/phagemid genomes (particles) produced by particular bacterial cell culture was calculated by determining the amount of phage ss DNA (from its absorbance) in 1 liter of overnight culture of particular bacterial strain containing ~ 10^12^ cells. We estimated that about 330 μg to l-1 of ssDNA is present in phage preparation of *N. gonorrhoeae* FA1090 and from 100 μg l-1 for E.coli and Salmonella strains up to 700 μg l-1 for *H. influenzae* (see Table S2) that correspond from about 1.5 x 10^13^ to 7.7 x 10^13^ phage-phagemid particles calculated according to equation: number of phage genomes = A x 6 x 10^17^/number of nucleotides in phage or phagemid particle, where A = OD269 – OD320.

1 ml of final phage preparation contained 10 mg of protein and 500 ng of ssDNA in 1 ul. The volume of phage suspension loaded on gel was 10 ul what corresponded to 100 ug of protein and 5 ug of ssDNA (line 3). This amount of ssDNA corresponds to 7.4 x 10^11^DNA molecules.

Proteins ( 10 ul equal 100 ug) separated on 4-15 % gel gave six bands of molecular size 35.0 kDa, 30 kDa, 26 kDa, 18 kDa, 12.5 kDa and 10 kDa. Use of Quantity one program allowed to calculated percent of weight of each band as: 34 %, 19 %, 12 %, 15 %, 14 % and 6 % and in turn the final weight as 34 ug, 19 µg, 12 µg, 15 µg, 14 µg and 6 µg. The number of each protein molecules per virion was calculated as the number of protein molecules in each band divided by the number of ssDNA molecules loaded on the gel.

1. Piekarowicz A, Klyz A, Majchrzak M, Adamczyk-Poplawska M, Maugel TK, Stein DC. Characterization of the dsDNA prophage sequences in the genome of *Neisseria gonorrhoeae* and visualization of productive bacteriophage. BMC Microbiol. 2007;7:66.
